# Influence of the Structure of Lattice Beams on Their Strength Properties

**DOI:** 10.3390/ma14195765

**Published:** 2021-10-02

**Authors:** Radosław Mirski, Łukasz Matwiej, Dorota Dziurka, Monika Chuda-Kowalska, Maciej Marecki, Bartosz Pałubicki, Tomasz Rogoziński

**Affiliations:** 1 Department of Wood-Based Materials, Poznań University of Life Sciences, 60-627 Poznań, Poland; radoslaw.mirski@up.poznan.pl (R.M.); macius.marecki@gmail.com (M.M.); 2 Department of Furniture Design, Poznań University of Life Sciences, 60-627 Poznań, Poland; lukasz.matwiej@up.poznan.pl (Ł.M.); tomasz.rogozinski@up.poznan.pl (T.R.); 3 Institute of Structural Analysis, Faculty of Civil and Transport Engineering, Poznań University of Technology, pl. Sklodowskiej-Curie 5, 60-965 Poznań, Poland; monika.chuda-kowalska@put.poznan.pl; 4 Department of Woodworking and Fundamentals of Machine Design, Poznań University of Life Sciences, 60-627 Poznań, Poland; bartosz.palubicki@up.poznan.pl

**Keywords:** eco-friendly wood, lattice beams, mechanical properties, bending strength

## Abstract

This paper presents the strength properties of wooden trusses. The proposed solutions may constitute an alternative to currently produced trusses, in cases when posts and cross braces are joined with flanges using punched metal plate fasteners. Glued carpentry joints, although requiring a more complicated manufacturing process, on the one hand promote a more rational utilisation of available structural timber resources, while on the other hand they restrict the use of metal fasteners. The results of the conducted analyses show that the proposed solutions at the current stage of research are characterised by an approx. 30% lower static bending strength compared to trusses manufactured using punched metal plate fasteners. However, these solutions make it possible to produce trusses with load-bearing capacities comparable to that of structural timber of grade C24 and stiffness slightly higher than that of lattice beams manufactured using punched metal plate fasteners. The strength of wooden trusses manufactured in the laboratory ranged from nearly 20 N/mm^2^ to over 32 N/mm^2^. Thus, satisfactory primary values for further work were obtained.

## 1. Introduction

Wooden trusses are an example of structural elements used in the construction industry—both in wooden and brick structures [1,2,3,4,5]. Trusses are members manufactured from several up to around a dozen planks joined to form triangular elements. They are often called truss girders or lattice beams. The chords may be parallel (called flat trusses), whereas in roof structures they are non-parallel. Chords are joined using diagonal and vertical members. An important feature of trusses, also referred to as lattice beams, is related to the elements (members) in truss nodes being connected so that only axial forces are present in those elements. In order to ensure such a distribution, the external load needs to be applied directly onto the truss nodes. Cross-sections of wooden truss members are typically 38 mm × 89 mm or 38 mm × 140 mm. Since the truss member cross-section is much smaller than its length, the effect of the structure dead weight on the level of internal forces is negligible. In such a situation, it may be assumed that only axial forces are present in truss members, whereas the effect of shear forces and bending moments from dead weight is negligible. It is the generally adopted convention that axial forces causing tension have the “+” sign (they are positive axial forces), while forces causing compression are assigned the “−“ sign (they are negative axial forces) [6,7]. 

**Figure 1:**
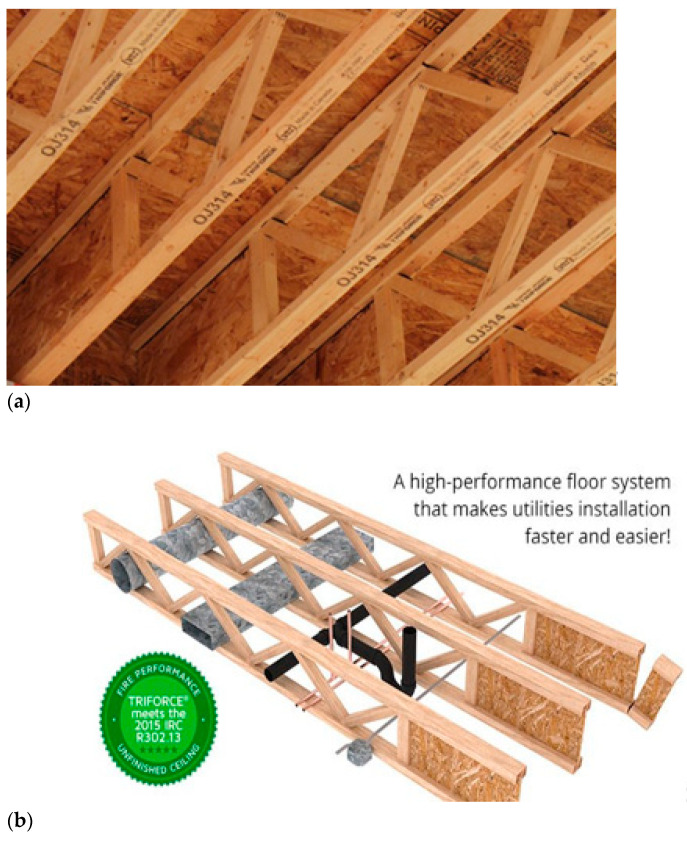
Examples of finger-jointed trusses: (**a**) roof constructions; (**b**) floor structures [38].

Relatively simple assumptions used to determine stresses found in truss members make it possible to apply various techniques and algorithms to optimise their shape [8,9,10,11,12,13,14,15]. The popularisation of numerical methods, as well as the availability of CAD software have facilitated the design process for systems based on lattice beams [16,17,18,19,20]. When punched metal plate fasteners appeared on the market in the 1950s, the process of wooden truss manufacture and design was considerably simplified. On the one hand, punched metal plate fasteners provide easy and fast connections of individual truss members, while on the other hand, dedicated computer programmes have been developed, defining plate size and timber cross-sections. The advantages of timber structures are related not only to the simplicity of their manufacture, but also to their durability, light weight, easy modification of shapes and other positive properties of timber itself [21,22,23,24]. Another advantageous feature of timber structures is connected to the reduction in the carbon footprint, thus having an essential environmental impact, particularly in view of the sustainable development concept [25,26]. A high ratio of stiffness and load-bearing capacity to the amount of used material is also stressed when talking about wooden trusses. Trusses may be manufactured both as elements of small dimensions and members of huge spans. They may be manufactured both from solid wood and glued laminated timber (glulam) [5,27,28]. Positive aspects of wooden trusses or timber itself may be eliminated as a result of an inappropriate connection of all structure components, which was presented in a specific and comprehensive manner in the AWC document [3]. The connection in the form of an articulated joint in individual truss members needs to be designed so as to carry the assumed load with no loss of system stiffness [27]. As it was mentioned above, it may be relatively easily attained with punched metal plate fasteners or previously used carpentry joints, but steel fasteners assembled using nails or screws may also be used for that purpose [29]. Nevertheless, punched metal plate fasteners seem to serve this purpose most effectively. Metal plate fasteners punched into timber provide greater strength and durability of truss members, since the arrangement of spikes in the plates, their number and their quality after an appropriate assembly provide an adequate fastening area, while the spikes themselves are anchored in timber and exhibit pull-out resistance [3,30,31,32,33]. A drawback of such a joint may result from the fact that truss cross-sections are increased so that a punched metal plate fastener with an adequate load-bearing capacity may be assembled (with an adequate cross-section area required). However, increasing the cross-section area of timber has some advantages, since it improves the fire resistance of a given element. A significant advantage of timber is connected with its strength, dependent only on the size of the destroyed cross-section and the load level, rather than the reduction in load-bearing capacity related to the increase in temperature [34,35,36]. In this approach, all steel elements in the wooden structure are typically considered to be more sensitive than timber itself. The results from extensive studies by Sultan [37] show that flat wooden lattice beams constituting the ceiling structure are characterised by comparable fire resistance, irrespective of the method, with which diagonals and chords are joined. This researcher investigated two types of trusses, one with punched metal plate fasteners and the other being finger-jointed trusses (Figure 1). 

Advantages of finger-jointed trusses include the considerable stiffness of such structures, potential application of cross diagonals and posts differing in cross-section areas from chords and easy manufacture, as well as the potential to adjust their length to specific needs on site (although the latter is only to a limited extent). 

In order to be able to vary the length more easily, the end of the truss often includes a chipboard strip. They can be OSB, fibreboards or other modern boards [39]. This is in line with the current trend of the use of eco-friendly wood-based panels in construction [40,41].

For all these reasons, it was decided to propose a similar solution, i.e., manufacturing flat trusses without the use of punched metal plate fasteners as elements at a greater risk of fire damage, requiring the use of timber of equal width for all members. The proposed solution, after being verified in further analysis, should facilitate the rational management of available timber resources.

## 2. Materials and Methods

It was decided to conduct the tests in two stages. In the first stage, the quality of the model lattice beam was evaluated, while in the second stage, the mechanical properties of actual model lattice beams were tested. 

The following assumptions for the structure and loading of lattice beams were adopted: 

Height: 240 mm,

Span—the distance between supports: 3240 mm,

Member cross-section: 38 mm × 60 mm,

External force (loading) (P) applied at (truss node): 2.78 kN. 

It was assumed that the quality of the designed lattice beams would not be lower than the strength of I-beams made with softwood lumber flanges. I-beams with a height of 240 mm are commonly used in single-family housing. Therefore, it was assumed that the designed lattice beams should be a substitute for these beams. Based on available materials (Steico, Czarnków, Poland; SWISS KRONO Żary, Poland), and taking into account the cross-sections of timber used for chords, it was calculated that the designed beams should carry a load, in a 4-point bending scheme, of min. 22.24 kN.

**Figure 2:**
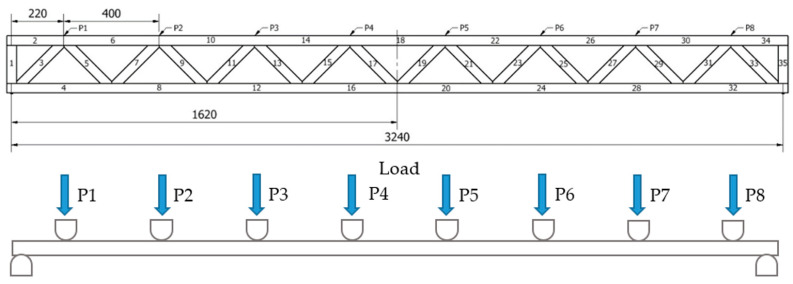
Truss loading scheme at each node (P_i_ = P = 2.78 kN)—scheme A.

**Figure 3:**
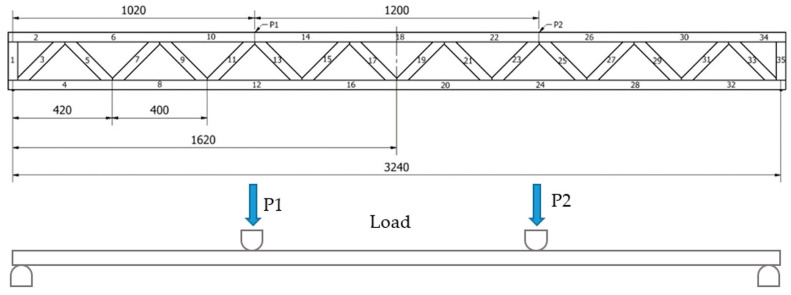
Truss loading diagram at two distinguished nodes (P_1_ = P_2_ = 11.12 kN )—scheme B.

Six bars of each type were tested (18 bars in total). All of the bars were burdened till destruction. To prevent any possible bar bucklings during bending in the structure of the machine, special vertical runners shifted 5 mm from the side surface of a bar were used. The bars were tested in two burdening schemes: scheme A (Figure 2) and scheme B (Figure 3). In scheme A, a burden of 22.24kN placed evenly and symmetrically in relation to the vertical axis on all 8 lattice knots (2.78kN each) was used. In scheme B, a burden of 22.24kN placed evenly and symmetrically in relation to the vertical axis on 2 selected lattice knots (11.12kN each) was used. During the defining of the terminal bar strength, scheme A was used (burden on all 8 lattice knots). The quality of the analysed lattice beams was tested in two loading schemes, i.e., applying force at each truss node and in two specified truss nodes. Loading points are presented in Figure 2 and Figure 3.

**Figure 4:**
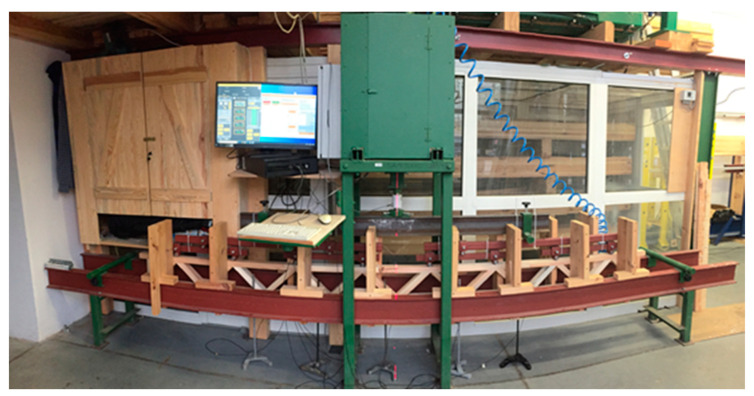
Picture of the test stand.

As shown in Figure 3, the second loading scheme may be considered a loading system at 4-point bending. The testing station for this loading system is presented in Figure 4. In addition to the force required to destroy the tested truss, the amount of deflection at a given force was also determined. Deflections were measured using dial indicators installed in 5 points below the deflection zone. 

**Figure 5:**
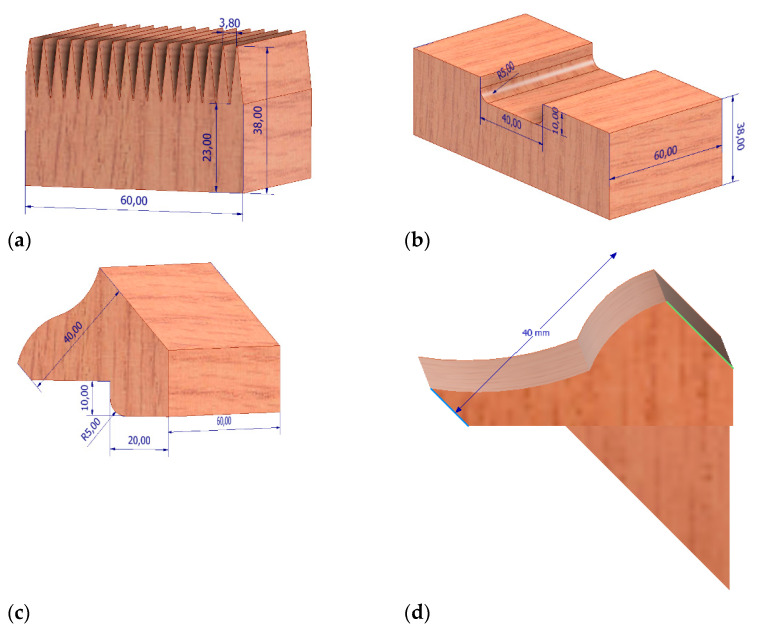
From the left: finger joint—chord of truss, UPP2 (**a**); tenon joint—chord of truss, tenon joint fragment of the lower belt UPP1 (**b**); tenon joint—diagonal member, tenon joint fragment of the crossbone UPP1 (**c**), tenon joint—diagonal member, tenon joint fragment of the crossbone UPP3 (**d**).

Deflection was determined using Sylva brand electronic dial sensors. The sensors were connected to a computer to discretely determine the deflection at a given load. Five sensors on laboratory racks were placed under the nodes (components shown in Figure 5). The middle sensor (no. 3) measured deflection at the centre of the beam span. In the tests of commercial lattice beams, two dial indicators were not placed directly under the truss nodes. Based on initial data on deflections provided by the linear regression function, deflections were determined for individual measurement points for pre-determined force values. Next, for the value equal to 1/300 length of the lattice beam, the value of force was established at which the deflection takes place (deflection at the centre of the span). Tests were carried out using testing machine type SAM75 (UPP, Poznań, Poland), which allows the movement of the crosshead with specified speed as well as by specified distance. The speed of crosshead movement was always constant and equal to 10 mm/min. To prevent loss of stability (buckling) of the beam during testing, special stops made of wood were used.

For the needs of laboratory analyses, three types of lattice beams were manufactured, with three specimens for each type. Both diagonals and posts were joined with chords using permanent carpentry joints prepared specifically for this project (mortise and tenon joints) by bonding the joined members. The polyurethane (PUR) adhesive was used in the joints. It is a single-component D4 adhesive, cross-linking when it comes into contact with moisture in the air. The two types of connection are presented in Figure 5. The third one may not be currently publicised, as it is patent pending. The presented lattice beams are denoted as UPP_x_: UPP_1_—mortise and tenon joint, UPP_2_—finger joint and UPP_3_—classified mortise and tenon design. The mortise and tenon joint was manufactured using CENTATEQ P-110 (HOMAG Group AG, Germany). In turn, the finger joint was produced using a spindle moulder by Felder with the FZK18NS180-002 cutter (Faba, Poland). The fourth type is lattice beams manufactured by the Witkowscy plant (Wieluń, Poland). They were conventional lattice beams with dimensions comparable to those of laboratory specimens. In this case, the elements were joined using punched metal plate fasteners. 

## 3. Results and Discussion

### 3.1. Calculation of Internal Axial Forces in Truss Members 

Table 1 presents values of internal axial forces found in individual lattice beam members. As shown by the data in Table 1, the greatest tensile forces are recorded in members no. 16 and 20, whereas the greatest compression force is found in member no. 18. In both cases, these values are 44.8 kN. In turn, in diagonals the greatest compression forces are recorded in members no. 3 and 33, while the greatest tensile forces are found in members no. 5 and 31. Compression forces in these diagonals amount to 15.84 kN, while tensile forces are 11.88 kN. Based on these values, Table 2 presents the calculated requirement for a specific timber grade needed to manufacture individual members of the designed lattice beam. For the analysed system, the critical strength value refers to the tensile strength of timber. At the assumed chord cross-section of 60 × 38 mm, to meet the assumed load-bearing conditions (strength equivalent to C24-grade timber or GL24h glulam), C35-grade timber is needed, according to EN 338 [42]. In turn, compressed chords may be manufactured from C22-grade timber, while diagonals may be made from out-of-grade C14 construction timber. In turn, when using C24 timber, the tensile chords need to be min. 78 mm × 40 mm in cross-section. 

Table 3 presents the distribution of internal axial forces in individual members in a situation, when the total loading of the lattice beam is identical, but this time the loads were applied only at two truss nodes (Figure 4—system B). In this case, each of the loads are equal to 11.12 kN. The greatest tensile force in the bottom chord is 55.6 kN, while in the diagonals it is 15.7 kN. As a result, irrespective of the scheme of loading and the assumed factors of safety, the required tensile strength of diagonals is approx. 3.5-fold lower than the required tensile strength of chords. As a consequence, this type of lattice beam may be optimised in terms of timber utilisation through the selection of timber quality/strength grades or appropriately adjusting the cross-section of these members.

Another important observation from these analyses is connected to the value of the force, with which the diagonals will be pulled out from the chord, when it works as the tensile member. The preliminary assumption that the diagonals are assembled at a 45° angle to chords simplifies the calculations, since they are reduced to one value. Thus, the force causing detachment of the diagonal from the chord amounts to 0.707 of the tensile force acting on the diagonal. As a result, for the first system (with each truss node being loaded) it is 8.4 kN and for the second system (two forces) it is 11.1 kN. This analysis shows that the strength of the glue line for the analysed PUR adhesive is min. 6 N/mm^2^ (mean 6.51 N/mm^2^). Thus, the surface area of the proposed joint should be min. 1400 mm^2^ or 1850 mm^2^ in order to transfer the loads in the first scheme (onto each truss node—scheme A) or in the second scheme (onto two specified nodes—scheme B). In mortise and tenon joints, the glue line area is min. 3300 mm^2^, while the glue line area for the finger joint is min. 21,000 mm^2^. 

### 3.2. Lattice Beam as a Solid Beam

Since relatively detailed static calculations may be made by replacing the spandrel beam/truss with a solid model, it was decided to introduce such transformations [43]. However, in this case, different stiffness values are used for such a model depending on the load acting on this system. In the analysed case, i.e., for loads acting on the lattice beam, the equivalent moment of inertia takes the form (1):

(1) $$J_{sec.}=\frac{J_{o}}{m_{1}}$$

where:

J_o_—moment of inertia of lattice beam chords at the centre of the span (2):

(2) $$J_{o}=e^{2}\frac{F_{g}\times F_{d}}{F_{g}+F_{d}}$$

where: 

e—theoretical height of the spandrel beam at the span centre, i.e., the dimension measured at the axes of gravity of chords;

F_d_—cross-section area of the bottom chords at the centre of its span;

F_g_—cross-section area of the top chords at the centre of its span;

m_1_– coefficients being the function of h_śr_/h_0_ (h_śr_—height of the spandrel beam at the centre of the span, h_0_—height of the spandrel beam at the support). For flat lattice beams, m_1_ = 1.

For the designed lattice beams, the following assumptions were made:

e = 202 mm; 

F_d_ = 2280 mm^2^;

F_g_ = 2280 mm^2^;

h_śr_/h_0_ = 1;

m_1_ = 1.

Thus, the equivalent moment of inertia for the designed lattice beams (UPP) is 4651.2 cm^2^, while for commercial lattice beams it is higher by almost 8%, i.e., 5017.6 cm^4^.

The index describing the bending strength of the cross-section is defined as the quotient of the second moment of area in relation to the principal axis of gravity of the cross-section and the distance to the most extreme fibres of the section (3):

(3) $$W_{z}=\frac{J}{\varepsilon_{max}}$$

where:

J—the principal moment of inertia of the cross-section in relation to axis z overlapping with the neutral axis of the cross-section; 

ε_max_—maximum distance of the most extreme fibres from the neutral axis.

Assuming that we deal with symmetric systems, the distance of the most extreme fibres is equal to a half of the lattice beam height. Table 4 presents the values of moments of inertia and cross-section strength indexes for the analysed lattice beam systems.

From the conducted analyses it can be seen that the bending strength of the model lattice beam for the 8-point loading scheme (A) is σ_8-p_ = 23.07 N/mm^2^, while for 2-point loading (4-point bending—scheme B) it is σ_2-p_ = 28.67 N/mm^2^. 

Since 4-point bending is the basic system used to evaluate the quality of structural elements, it needs to be stated that properties obtained in this test are more reliable when comparing structural elements differing in their structure. Nevertheless, we are aware that the first analysed scheme (A) is closer to the real situation, and is thus more reliable for the use of trusses as structural elements. Yet, this may not be readily referred to the numerous publications discussing beams as structural elements. Moreover, the authors also intended to relate the results to members manufactured from solid wood. For this reason, when referring the designed lattice beams to construction timber of grade C24 (most commonly used in practice), it needs to be stated that the adopted preliminary values are comparable to those of this timber grade. When analysing the obtained results in more detail, the designed lattice beams should thus have the bending moment in a 4-point bending test greater than 9.31 kN m, or the force required for the failure of the lattice beam should exceed 18.62 kN. In such a case, the designed systems will exhibit strength comparable to that of a solid timber beam.

### 3.3. Analysis of Laboratory Testing Results

Table 5 presents the mechanical properties of lattice beam models evaluated in the 4-point bending test. The preliminary assessment of the potential applicability of the manufactured lattice beams as an alternative to lattice beams using punched metal plate fasteners indicates that only those produced with finger joints of chords with diagonals are characterised by comparable mechanical properties. Lattice beams manufactured by joining diagonals with chords using mortise and tenon joints exhibited an approx. 30% lower strength. In the case of mortise and tenon joints, UPP_1_ failure was observed typically in timber within the truss joint. 

**Figure 6:**
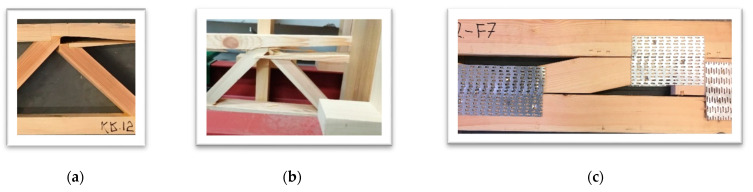
Failure images of tested lattice beams: (**a**) UPP1; (**b**) UPP2; (**c**) industrial.

Depending on the annual growth increment, systems cracking was observed at the transition zone from early wood to late wood (Figure 6a). In turn, in the case of the finger joint, failure was recorded in the most extreme truss joint, and it consisted of a shearing of the finger joint (Figure 6b). In UPP_3_ lattice beams, failure was observed in the tensile chord. However, it was at the finger joint connecting the chord member lengthwise. In the case of commercial lattice beams, in the strength testing, failure was observed either in the timber of the tensile chord, or the punched metal plate fasteners were destroyed or detached. This failure type is shown in Figure 6c. Due to the too-low image-recording frequency of the camera used to record the tests, in many cases it was difficult to assess (or estimate) which failure mechanism initiated the destruction of the beam. Lattice beams manufactured at the laboratory are characterised by a slightly lower moisture content than commercial lattice beams (7–8%). To a certain extent, this may be reflected in the stiffness of the analysed specimens, which will decrease with an increase in wood moisture content.

Another significant criterion for frame systems is connected with the volume of deflections appearing at loading. Admissible deflections in the span and the bracket are determined following the PN-EN 1995-1-1:2010 standard [44], while for floor beams admissible deflections amount to 1/300 of their length (L/300). The values of deflections and bending moments at such deflections are given in Table 6. As shown by the presented data, the most advantageous results are obtained for the lattice beam, in which chords were joined with diagonals using a carpentry joint designed specifically for the purpose of this project, i.e., the longitudinal mortise and tenon joint. 

While this is an improvement by only 10% compared to the lattice beam manufactured with members joined using punched metal plate fasteners, it needs to be remembered that this novel solution is still being modified. What is surprising is that the lowest stiffness was recorded for lattice beams, in which chords and diagonals were connected using finger joints. Thus, these lattice beams are characterised by a considerable strength, while at the same time they are highly susceptible to load-induced strain, which probably results from the moulding of finger joints along the entire chord length. 

The results obtained are similar to those obtained by other research centres [45,46]. The comparison itself is relatively difficult. Very often, for simplicity, other studies do not maintain the length/height relationship and the trusses are tested in 3-point bending. However, the authors mentioned above indicate that their trusses fail under loads of 6–8 kN to 11–14 kN. Importantly, these authors indicate that glued trusses exhibit superior properties to nailed trusses.

Table 7 presents testing results from a lattice beam UPP_3_ loaded at each truss node (scheme A). As shown by the data, deflection amounting to 1/300 length occurs at the bending moment of 3.77 kN·m. In turn, the ultimate bending moment is as high as 9.53 kN·m. The strength of the analysed lattice beam referred to as a solid beam amounts to more than 24 N/mm^2^. Strength, even at a relatively low moisture content of used timber, is relatively high. Moreover, the mean value of the load applied at each truss node is 2.91 kN and, thus, it is only approx. 5% higher than the one assumed for calculations for the model lattice beam. 

## 4. Conclusions

It results from these analyses that it is feasible to manufacture lattice beams with parallel chords to serve as truss beams, when punched metal plate fasteners joining chords with diagonals are replaced by carpentry joints. Preliminary theoretical assumptions were correctly selected and thus provide grounds for design work on such lattice beams. Static analysis showed that cross-sections of elements used as chord fasteners (diagonals, posts) may be considerably reduced or made from inferior-grade timber. Of the two proposed solutions, i.e., perpendicular and longitudinal, more advantageous results were obtained for the longitudinal joint. Moreover, it results from the conducted tests that: 

The greatest load-bearing capacity, determined in the bending test, is found for lattice beams manufactured using punched metal plate fasteners; 

Only a slightly lower stiffness, amounting to 32.2 N/mm^2^, was recorded for lattice beams, elements of which were connected using finger joints; however, they show deflection of 1/300 length even under the smallest load;

Lattice beams, in which chords were connected with diagonals using glued carpentry joints (UPP3), exhibit an approx. 35% lower bending strength, although they show much smaller deflections under the same loading compared to lattice beams manufactured using finger joints.

Although neither of the joints met the requirements in terms of the assumed load-bearing capacity, in view of the observed failure mechanism, it was decided that in further stages of study the mortise and tenon joints UPP_3_ will be developed. In further studies, it is intended to conduct tests on solid timber chords and to a certain extent modify the shape of the joint itself.

The results of the study provide a valuable basis for further design work. Developers especially are looking for cheaper alternatives to both solid wood and glued laminated timber. Additionally, single-family houses, often in Central Europe, realized in an economic way, are looking for cheaper and at the same time easy-to-install construction materials.

## Figures and Tables

**Table 1:** Values of forces in bars—scheme A (Figure 2).

No. Member	Force (kN)	Type of Member	No. Member	Force (kN)	Type of Member
1	0.00	s	10	−33.6	tc
2	0.00	pg	11	−7.92	dm
3	−15.84	k	12	39.2	bc
4	11.20	pd	13	3.96	dm
5	11.88	s	14	−42.00	tc
6	−19.60	pg	15	−3.96	dm
7	−11.88	k	16	44.80	bc
8	28.00	pd	17	0.00	dm
9	7.92	s	18	−44.8	tc

s—bar; pg—lower belt; pg—upper belt; k—crossbone.

**Table 2:** The minimum strength.

No. Member	Force (kN)	Type of Member	f_c/t_ * (N/mm^2^)	Min. Class of Timber	*f_c/t_* by Standard ** (N/mm^2^)
16	44.80	pd	19.65	C35	21
18	−44.80	pg	19.65	C22	20
3	−15.84	s	6.95	C14	16
5	11.88	s	5.21	C14	8

* compression strength/tensile strength, ** EN 338 [42].

**Table 3:** Values of forces in bars—scheme B (Figure 3).

No. Member	Force (kN)	Type of Member	No. Member	Force (kN)	Type of Member
1	0.00	s	10	−44.45	pg
2	0.00	pg	11	−15.72	k
3	11.12	k	12	55.57	pg
4	−15.72	pd	13	0.00	k
5	15.72	k	14	−55.57	pg
6	−22.23	pg	15	0.00	k
7	−15.72	k	16	55.57	pg
8	33.35	pg	17	0.00	k
9	15.72	k	18	−55.57	pg

s—bar; pg—lower belt; pg—upper belt; k—crossbone.

**Table 4:** Results for trusses.

Type of Truss	Height (mm)	J_sec._ (cm^4^)	W_z_ (cm^3^)	U_max_ (mm) *
UPP	240	4651.2	387.64	10.8
Witkowski	240	5017.6	418.13	10.8

* allowable deflection/maximum permissible deflection—L/300; L—length of the truss.

**Table 5:** Evaluation of the mechanical properties of the manufactured truss models.

Joint Type	M_g max_ (kN·m)	*f_m_* (N/mm^2^)
UPP_1_	7.67 (0.82) *	19.79
UPP_2_	12.48 (1.39)	32.18
UPP_3_	8.32 (0.33)	21.42
Steel truss plates	14.06 (1.34)	33.62

* SD (standard deviation).

**Table 6:** Evaluation of the mechanical properties of manufactured models of lattice beams.

Connection Type	Sensor Number					M_g003_ * (kN·m)	M_g max_ (kN·m)
	1	2	3	4	5		
	Deflection Value (mm)						
Tenons UPP_1_	−8.02	−10.14	−10.68	−10.42	−8.45	5.11 (0.30) **	7.67
Finger joints UPP_2_	−4.85	−8.42	−10.73	−9.04	−8.86	4.16 (0.26)	12.48
Tenons UPP_3_	−7.69	−9.57	−10.76	−9.76	−7.80	5.65 (0.32)	8.32
Barbed plates	−6.46	−9.19	−11.00	−9.38	−6.97	5.18 (0.16)	14.06

* moment at deflection to equal 1/300; ** SD (standard deviation).

**Table 7:** Physical and mechanical properties of UPP3 lattice beam loaded at 8 points.

Typ	Mg003 * (kN·m)	Mgmax (kN·m)	F (kN)	fm (N/mm^2^)	MC ** (%)
UPP3	3.77 (0.14)	9.53 (0.65)	2.91	24.58	6.7

* bending moment at 1/300; ** wood moisture content.

## Data Availability

The data presented in this study are available on request from the corresponding author.
